# Soy isoflavones inducing overt hypothyroidism in a patient with chronic lymphocytic thyroiditis: a case report

**DOI:** 10.1186/s13256-017-1418-9

**Published:** 2017-09-05

**Authors:** Yuya Nakamura, Isao Ohsawa, Yoshikazu Goto, Mayumi Tsuji, Tatsunori Oguchi, Naoki Sato, Yuji Kiuchi, Motonori Fukumura, Masahiro Inagaki, Hiromichi Gotoh

**Affiliations:** 1 Department of Internal Medicine, Saiyu Soka Hospital, 1-7-22 Matsubara, Soka City, Saitama 344-0041 Japan; 2 Department of Pharmacology, School of Medicine, Showa University, Shinagawa-ku, Tokyo, Japan; 3 Division of Natural Medicine and Therapeutics, Department of Clinical Pharmacy, School of Pharmacy, Showa University, Shinagawa-ku, Tokyo, Japan; 4 Department of Chemistry, College of Arts and Sciences, Showa University, Fujiyoshida City, Yamanashi Japan

**Keywords:** Isoflavones, Soybean, Barley young leaf, kale, Hypothyroidism, Chronic lymphocytic thyroiditis, Thin-layer chromatography

## Abstract

**Background:**

Many people have thyroid conditions that make them susceptible to hypothyroidism. If the foods they eat may interfere with the production of thyroid hormone, which can lead to development of serious hypothyroidism. The danger of health drinks should always be noted.

**Case presentation:**

A 72-year-old Japanese woman was previously diagnosed with chronic lymphocytic thyroiditis caused by a goiter and had an elevated thyroid-stimulating hormone level (6.56 μIU/ml), a high anti-thyroid peroxidase antibody level (>600 IU/ml), and a high antithyroglobulin level (> 4000 IU/ml) but normal levels of free triiodothyronine (3.08 pg/ml) and thyroxine (1.18 ng/ml). She presented to our hospital with sudden-onset general malaise, edema, and hoarseness with an elevated thyroid-stimulating hormone (373.3 μIU/ml) level and very low triiodothyronine (< 0.26 pg/ml) and thyroxine (0.10 ng/ml) levels. It was determined that for 6 months she had been consuming a processed, solved health drink (“barley young leaf”) in amounts of 9 g/day, which included soybean and kale powder extract. Hypothyroidism might be affected by ingredients of health drinks. She discontinued consumption of the health drink immediately and began taking 12.5 μg of levothyroxine. The amount of levothyroxine was gradually increased every 3 days up to 100 μg. At day 61, her thyroid-stimulating hormone level had decreased (6.12 μIU/ml), her free triiodothyronine (2.69 pg/ml) and thyroxine (1.56 ng/ml) levels had increased, and her general condition was improved. Among risky foods lowering thyroid function, some experimental studies have revealed that isoflavones reduce thyroid function. Therefore, we measured the presence of isoflavones in the patient’s frozen serum with thin-layer chromatography. After she discontinued consumption of the health drink, two components quickly disappeared, and the other three components gradually decreased. On the basis of developing solvent composition and a positive ferric chloride reaction in thin-layer chromatography experiment, the five ingredients that disappeared or decreased were highly suspected to be soy isoflavones.

**Conclusions:**

This case emphasizes that consuming health drinks that include soy isoflavone powder extracts can lead to severe hypothyroidism.

## Background

Many people have thyroid conditions that make them susceptible to hypothyroidism. For example, 10% of a disease-free population was reported to have positive results for anti-thyroid peroxidase antibodies (TPOAb) or antithyroglobulin (anti-Tg) [1], indicating that many patients have potentially chronic lymphocytic thyroiditis. However, there are many exogenous foods influencing the thyroid. If the foods interfere with the production of thyroid hormone, they can cause serious hypothyroidism.

We report a case of a patient with severe hypothyroidism induced by consuming a health drink. To the best of our knowledge, there are almost no other reports of patients with severe hypothyroidism induced by isoflavone. The components of the substances suspected to be isoflavones disappeared or decreased along with the clinical impairment of our patient. To the best of our knowledge, this is the first report of the presence of isoflavone in the serum of a patient with severe hypothyroidism.

## Case presentation

A 72-year-old Japanese woman had been followed because of chronic lymphocytic thyroiditis caused by a goiter. She had an elevated thyroid-stimulating hormone (TSH) level (6.56 μIU/ml), a high TPOAb level (> 600 IU/ml), and a high anti-Tg level (> 4000 IU/ml) but normal levels of free triiodothyronine (T3; 3.08 pg/ml) and thyroxine (T4; 1.18 ng/ml). Her past medical history included only hypertension. She did not have any fever or neck pain suggestive of subacute or painless thyroiditis during recent months, and she had not taken any medication that would reduce thyroid function. Her social, family, and environmental histories were also unrevealing.

**Fig. 1:**
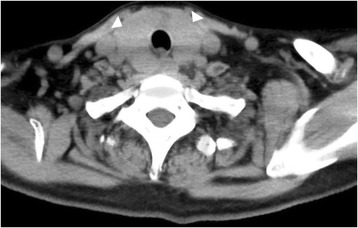
Computed tomographic scan showing thyroid enlargement (*arrowheads*)

**Fig. 2:**
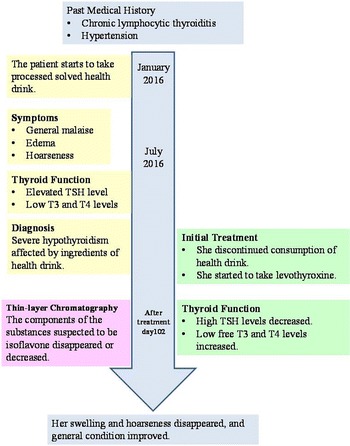
Timeline

The patient had been taking a processed, solved health drink (“barley young leaf”) in amounts of 9 g/day, which included soybean and kale powder extract, for 6 months. She had gradually developed general malaise, edema, and hoarseness. She presented to our hospital with sudden-onset elevated TSH (373.3 μIU/ml) level and very low T3 (< 0.26 pg/ml) and T4 (0.10 ng/ml) levels. Her levels of total cholesterol, triglyceride, lactate dehydrogenase, aspartate transaminase, creatine phosphokinase, and immunoglobulin G were increased. These laboratory findings are shown in Table 1. Her goiter was swollen with mild hardness; her blood pressure at admission was 123/77 mmHg; her body temperature was 36.0 °C; and her pulse rate was 55 beats/minute. Computed tomography (Fig. 1) and thyroid ultrasound revealed bilateral thyroid enlargement. In addition, her uptake rate of ^99m^Tc-pertechnetate was reduced. On the basis of these findings, we diagnosed severe hypothyroidism affected by ingredients of the health drink. She discontinued consumption of the health drink immediately and began taking 12.5 μg of levothyroxine. The amount of levothyroxine was gradually increased every 3 days up to 100 μg. After oral administration of levothyroxine, her high TSH levels decreased gradually, and her low free T3 and T4 levels increased. Her swelling and hoarseness disappeared, and her general condition improved. Her free T3 and T4 levels did not decrease in the follow-up period of the subsequent 6 months. The time line of this patient’s clinical course is shown in Fig. 2. Her TSH, free T3, and T4 levels are shown in Table 2.

**Table 1:** Patient’s laboratory findings on admission

	Values	Normal ranges	Blood chemistry	Values	Normal ranges
Blood cell counts					
WBC, *n*/μl	57×10^2^	40–90×10^2^	TP, g/dl	8.3	6.7–8.3
RBC, *n*/μl	389×10^4^	380–480×10^4^	Alb, g/dl	4.0	3.9–4.9
Hb, g/dl	11.3	12.0–15.2	BUN, mg/dl	13.2	8.0–22.0
Hct, %	36.0	35–48	Cr, mg/dl	1.0	0.4–0.7
Plt, *n*/μl	13.1×10^4^	14–34×10^4^	UA, mg/dl	4.9	3.0–5.5
			Na^+^, mEq/L	141	135–147
Serological tests			K^+^, mEq/L	3.5	3.5–5.0
Fe, μg/dl	58	43–172	Cl^−^, mEq/L	102	98–108
UIBC, μg/dl	213	137–325	Ca^2+^, mg/dl	9.2	8.8–10.2
TIBC, μg/dl	271	251–398	Pi, mg/dl	3.2	2.5–4.5
Ferritin, ng/ml	72.8	5–157	AST, U/L	41	13–33
IgG, mg/dl	2231	1156	ALT, U/L	28	6–27
IgA, mg/dl	397	103	γ-GTP, U/L	31	10–47
IgM, mg/dl	128	125	ALP, U/L	208	115–359
NT-proBNP, pg/ml	49	< 125	LDH, U/L	330	119–229
			T-Bil, mg/dl	0.6	0.2–1.2
Urinalysis			CPK, U/L	567	45–163
Protein	–	–	T-chol, mg/dl	310	130–220
Occult blood test	–	–	TG, mg/dl	233	30–150
			HDL-chol, mg/dl	54	41.5–67.3
Fecal occult blood test	–	–	LDL-chol, mg/dl	187	70–139
			Glu, mg/dl	85	70–110
			CRP, mg/dl	0.15	< 0.30

*Abbreviations: Alb* Albumin, *ALP* Alkaline phosphatase, *ALT* Alanine transaminase, *AST* Aspartate transaminase, *BUN* Blood urea nitrogen, *Ca* ^*2+*^ Calcium, *Cl* ^*−*^ Chloride, *CPK* Creatine phosphokinase, *Cr* Creatinine, *CRP* C-reactive protein, *Fe* Iron, *γ-GTP* γ-Glutamyltransferase, *Glu* Glucose, *Hb* Hemoglobin, *Hct* Hematocrit, *HDL-chol* High-density lipoprotein cholesterol, *IgA* Immunoglobulin A, *IgG* Immunoglobulin G, *IgM* Immunoglobulin M, *K* ^+^ Potassium, *LDH* Lactate dehydrogenase, *LDL-chol* Low-density lipoprotein cholesterol, *Na* ^+^ Sodium, *NT-proBNP* N-terminal pro-brain natriuretic peptide, *Pi* Inorganic phosphate, *Plt* Blood platelets, *RBC* Red blood cells, *T-Bil* Total bilirubin, *T-chol* Total cholesterol, *T-chol* Total cholesterol, *TG* Triglyceride, *TIBC* Total iron-binding capacity, *TP* Total protein, *TSAT* Transferrin saturation, *UA* Uric acid, *UIBC* Unsaturated iron-binding capacity, *WBC* White blood cell

**Table 2:** Patient’s clinical course based on laboratory findings after discharge

	Before treatment	Day 11	Day 33	Day 61	Day 102	Normal range
TSH, μIU/ml	373.7	341.3	40.66	6.12	11.68	0.50–5.00
fT3, pg/ml	< 0.26	0.55	2.47	2.69	2.58	2.30–4.00
fT4, ng ml	0.1	0.28	1.41	1.56	1.45	0.90–1.70

*Abbreviations: fT3* Free triiodothyronine, *fT4* Thyroxine, *TSH* Thyroid-stimulating hormone

**Fig. 3:**
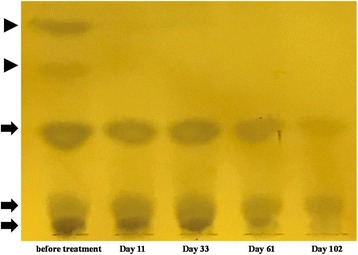
Results of the thin-layer chromatography. After discontinuing health drink, two components (arrow heads) quickly disappeared and the other three components (arrows) gradually decreased. From the composition of the developing solvent and the positive findings of ferric chloride, these ingredients were speculated highly likely for soy isoflavone

There are many risky foods lowering thyroid function, such as soybean and cruciferous vegetables, especially in a patient with chronic lymphocytic thyroiditis. Among these foods, some experimental studies have shown that isoflavone reduces thyroid function [2, 3]. It was suspected that isoflavones might be the reason for our patient’s hypothyroidism. Therefore, we measured the presence of isoflavone in the patient’s frozen serum at 5 points from before admission to day 102 for further investigation. A blood sample was pretreated as described previously [4]. The sample was analyzed with thin-layer chromatography on precoated silica gel 60 F_254_ or RP-18 WF_254_ plates (Merck Millipore Corporation, Darmstadt, Germany), with detection achieved by spraying with iron(III) chloride solution. After she discontinued consuming the health drink, two components quickly disappeared, and the other three components gradually decreased (Fig. 3). On the basis of developing solvent composition and a positive ferric chloride reaction in thin-layer chromatography experiment, the five ingredients that disappeared or decreased were highly suspected to be soy isoflavones.

## Discussion

Isoflavone, one of the isoflavonoids, is associated with breast cancer, prostate cancer, cerebral infarction, and myocardial infarction [5–7]. Moreover, some researchers have reported that soy isoflavone might reduce thyroid function by suppressing thyroid peroxidase [2, 3]. In our patient, the components of the substances suspected to be isoflavones had disappeared or decreased along with her clinical impairment. To the best of our knowledge, this is the first case report in which the presence of isoflavone has been examined in the serum of a patient with severe hypothyroidism. This point is unique; to the best of our knowledge, there have been almost no other reports in which the presence of isoflavone has been examined in patient serum. The other following two mechanisms of hypothyroidism by soybean are considered: (1) An alcohol-soluble component in soybean inhibits iodide uptake [8], and (2) phytic acid salt in soybean chelates the essential minerals, especially zinc, for thyroid hormone production [9, 10]. Our patient presented with severe hypothyroidism after 6 months of regular consumption of a health drink. Because the thyroid contains several months’ storage of thyroid hormone [11], overt hypothyroidism might present late. It is necessary to investigate further the detailed mechanisms of hypothyroidism caused by soy isoflavones.

## Conclusions

This case emphasizes that consuming health drinks that include soy isoflavone powder extract can lead to severe hypothyroidism.

## References

[1] Hollowell JG, Staehling NW, Flanders WD, Hannon WH, Gunter EW, Spencer CA, *et al.* Serum TSH, T_4_, and thyroid antibodies in the United States population (1988 to 1994): National Health and Nutrition Examination Survey (NHANES III). J Clin Endocrinol Metab. 2002;87:489–99. 10.1210/jcem.87.2.8182 11836274

[2] Chang HC, Doerge DR. Dietary genistein inactivates rat thyroid peroxidase in vivo without an apparent hypothyroid effect. Toxicol Appl Pharmacol. 2000;168:244–52. 10.1006/taap.2000.9019 11042097

[3] Munro IC, Harwood M, Hlywka JJ, Stephen AM, Doull J, Flamm WG, *et al.* Soy isoflavones: a safety review. Nutr Rev. 2003;61:1–33. 10.1301/nr.2003.janr.1-33 12638461

[4] Kaneko H, Yamato Y, Teramura Y, Fujiwara T, Suzuki A, Kawamura A, *et al.* Metabolites of micafungin in rats and dogs. Jpn J Chemother. 2002;50:88–93.

[5] Yamamoto S, Sobue T, Kobayashi M, Sasaki S, Tsugane S. Japan Public Health Center-Based Prospective Study on Cancer Cardiovascular Diseases Group. Soy, isoflavones, and breast cancer risk in Japan. J Natl Cancer Inst. 2003;95:906–13.

[6] Kokubo Y, Iso H, Ishihara J, Okada K, Inoue M, Tsugane S, JPHC Study Group. Association of dietary intake of soy, beans, and isoflavones with risk of cerebral and myocardial infarctions in Japanese populations: the Japan Public Health Center-based (JPHC) study cohort I. Circulation. 2007;116:2553–62. 10.1161/CIRCULATIONAHA.106.683755 18025534

[7] Kurahashi N, Iwasaki M, Sasazuki S, Otani T, Inoue M, Tsugane S, Japan Public Health Center-Based Prospective Study Group. Soy product and isoflavone consumption in relation to prostate cancer in Japanese men. Cancer Epidemiol Biomarkers Prev. 2007;16:538–45. 10.1158/1055-9965.EPI-06-0517 17337648

[8] Tran L, Hammuda M, Wood C, Xiao CW. Soy extracts suppressed iodine uptake and stimulated the production of autoimmunogen in rat thyrocytes. Exp Biol Med (Maywood). 2013;238:623–30. 10.1177/1535370213489488 23918874

[9] Zhou JR, Erdman Jr JW. Phytic acid in health and disease. Crit Rev Food Sci Nutr. 1995;35:495–508. 10.1080/10408399509527712 8777015

[10] Szkudelski T. Phytic acid-induced metabolic changes in the rat. J Anim Physiol Anim Nutr (Berl). 2005;89:397–402. 10.1111/j.1439-0396.2005.00532.x 16401191

[11] Ingbar SH, Woeber KA. The thyroid gland. In: Williams RH, editor. Textbook of endocrinology. 6th ed. New York: Saunders; 1981. p. 117–247.

